# Antiproliferative and Apoptotic Effects Triggered by Grape Seed Extract (GSE) *versus* Epigallocatechin and Procyanidins on Colon Cancer Cell Lines

**DOI:** 10.3390/ijms13010651

**Published:** 2012-01-10

**Authors:** Simona Dinicola, Alessandra Cucina, Alessia Pasqualato, Fabrizio D’Anselmi, Sara Proietti, Elisabetta Lisi, Gabriella Pasqua, Donato Antonacci, Mariano Bizzarri

**Affiliations:** 1 Department of Experimental Medicine, “La Sapienza” University, Viale Regina Elena 324, Rome 00161, Italy; E-Mails: simona.dinicola@uniroma1.it (S.D.); sara.proietti@uniroma1.it (S.P.); lisi.elisabetta@gmail.com (E.L.); 2 Department of Surgery ‘Pietro Valdoni’, “La Sapienza” University, Via A. Scarpa 14, Rome 00161, Italy; E-Mails: alessandra.cucina@uniroma1.it (A.C.); fabrizio.danselmi@uniroma1.it (F.D.); 3 Department of Neuroscience and Imaging, “G. D’Annunzio” University, Via dei Vestini 29, Chieti Scalo 66013, Italy; E-Mail: alessia.pasqualato@live.it; 4 Italian Space Agency, Viale Liegi 26, Rome 00198, Italy; 5 Department of Environmental Biology, “La Sapienza” University, Piazzale Aldo Moro 5, Rome 00185, Italy; E-Mail: gabriella.pasqua@uniroma1.it; 6 CRA (Agricultural Research Council), Via Casamassima 148, Turi (Bari) 70010, Italy; E-Mail: donato.antonacci@entecra.it

**Keywords:** apoptosis, Caco2, HCT-8

## Abstract

Grape seed extract has been proven to exert anticancer effects on different tumors. These effects are mainly ascribed to catechin and procyanidin content. Analytical studies demonstrated that grape seed extract composition is complex and it is likely other components could exert biological activities. Using cell count and flow cytometry assays, we evaluated the cytostatic and apoptotic effects produced by three different grape seed extracts from *Italia*, *Palieri* and *Red Globe* cultivars, on Caco2 and HCT-8 colon cancer cells. These effects were compared to those induced by epigallocatechin and procyanidins, alone or in association, on the same cell lines. All the extracts induced growth inhibition and apoptosis in Caco2 and HCT-8 cells, along the intrinsic apoptotic pathway. On both cell lines, growth inhibition induced by *Italia* and *Palieri* grape seed extracts was significantly higher than that it has been recorded with epigallocatechin, procyanidins and their association. In Caco2 cells, the extract from *Red Globe* cultivar was less effective in inducing growth inhibition than procyanidins alone and in association with epigallocatechin, whereas, in HCT-8 cells, only the association of epigallocatechin and procyanidins triggers a significant proliferation decrease. On both cell lines, apoptosis induced by *Italia*, *Palieri* and *Red Globe* grape seed extracts was considerably higher than has been recorded with epigallocatechin, procyanidins and their association. These data support the hypothesis by which other compounds, present in the grape seed extracts, are likely to enhance the anticancer effects.

## 1. Introduction

Epidemiologic studies have demonstrated that the consumption of a vegetables and fruits based diet significantly reduces the overall cancer risk [1,2]. As a proof-of-concept, the high-fat low-vegetables based diets are thought to be a major cancer risk factor [3]. Indeed, the estimate of the cancer risk for diet exceeded that one for tobacco and infections [4]. Fruits and vegetables have been hypothesized to be major dietary contributors to cancer prevention because they are potentially full of anticancer substances [5]. Consequently, in recent years some studies attempted to isolate and characterize potential chemopreventive agents present in fruits and vegetables. In this regard, many phytochemicals of different chemical nature, such as catechins, bioflavonoids, proanthocyanidins and phyto-estrogens, have revealed promising chemopreventive and/or anticancer efficacy in several cell cultures and animal models [6–11]. In particular, substances extracted from grape seeds (flavan-3-ols, catechins, epigallocatechin, procyanidins) have demonstrated to exert numerous and different biological effects in several cancer types, both *in vitro* and *in vivo* [12–15]. Anticancer effects exerted by grape seed extract (GSE) are generally attributed to the epigallocatechin and procyanidin content; indeed, in cancer cells cultures epigallocatechin and procyanidins share relevant pro-apoptotic as well as growth inhibitory properties [16,17]. However, analytical studies demonstrated that GSE composition is highly complex [18] and therefore it is likely that anticancer effects exerted by GSE cannot be reduced only to its epigallocatechin and procyanidin content.

We evaluated the cytostatic and apoptotic effects produced by three different grape seed extracts from *Italia*, *Palieri* and *Red Globe* cultivars on Caco2 and HCT-8 colon cancer cells. These effects were compared to those ones induced by epigallocathechin and procyanidins, alone or in association, on the same cell lines. Commercially available epigallocathechin (EGC) and grape seed procyanidins (GSP) were used at the same concentration they have in the GSE of each cultivar.

## 2. Results and Discussion

### 2.1. Grape Seed Extract

Composition of *Italia*, *Palieri* and *Red Globe* GSE was determined as previously reported [18]. The three cultivars show significant differences with respect to their composition: *Italia* GSE contains 2.5 mg/g catechins and 4.1 mg/g procyanidins; *Palieri* GSE contains 6.2 mg/g catechins and 5.6 mg/g procyanidins; *Red Globe* GSE contains 3.9 mg/g catechins and 3.9 mg/g procyanidins.

### 2.2. GSE-Induced Growth Inhibition

**Figure 1:**
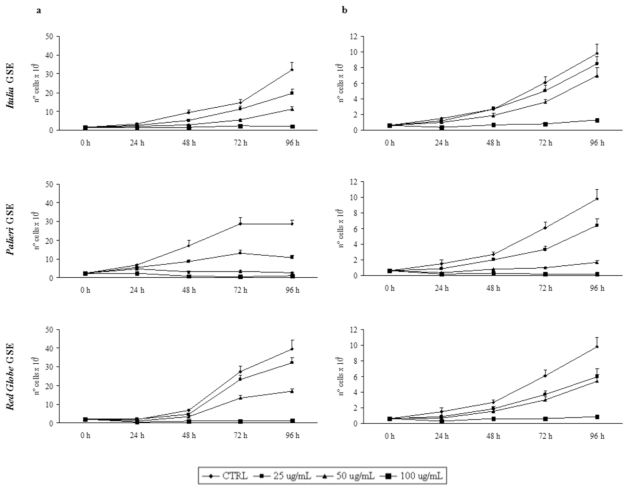
Grape seed extract (GSE)-induced growth inhibition. (**a**) Effects of GSE on proliferation of Caco2 and (**b**) HCT-8 cells after 24, 48, 72 and 96 hours. The cells were stimulated with 25, 50 and 100 μg/mL of *Italia*, *Palieri* and *Red Globe* GSE. Results are the mean ± SD of six independent experiments performed in duplicate.

Growth inhibition was recorded every 24 hours, until 96 hours. GSE did not induce any detectable growth inhibitory effect on cultured human fibroblasts (data not shown). Proliferation rates of Caco2 and HCT-8 cells were otherwise significantly reduced by GSE in a dose- and time-dependent manner in respect to untreated control cells (Figure 1a, b).

In both cell lines treated with GSE obtained from *Italia*, *Palieri* and *Red Globe* cultivars, growth rates displayed a similar trend. In Caco2 cells, treatment with *Italia* and *Palieri* GSE induced a statistically significant growth inhibition at each GSE concentration and at all the times considered (*p* < 0.01), reaching the most relevant inhibition after 96 hours at 50 and 100 μg/mL (*p* < 0.001). Otherwise, in *Red Globe*-treated Caco2 cells the growth inhibition is statistically significant at all the times which it was studied only at 50 and 100 μg/mL (*p* < 0.001).

In HCT-8 cells, treatment with *Italia* GSE induced a statistically significant growth inhibition only at 50 μg/mL (*p* < 0.01) and 100 μg/mL (*p* < 0.001) at all the times considered. Nevertheless, in *Palieri* and *Red Globe*-treated HCT-8 cells the growth inhibition is statistically significant starting from 48 hours for all GSE concentrations (*p* < 0.001).

### 2.3. Epigallocatechin and Procyanidin-Induced Growth Inhibition

**Figure 2:**
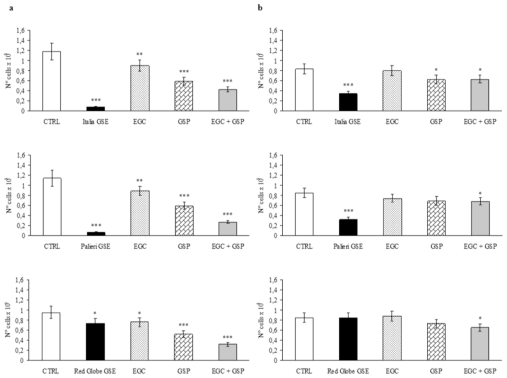
Epigallocathechin (EGC) and grape seed procyanidins (GSP)-induced growth inhibition *versus* GSE. (**a**) Effects of commercially available EGC and GSP at the concentration they have in 100 μg/mL of GSE, obtained from *Italia*, *Palieri* and *Red Globe* cultivars on proliferation of Caco2 and (**b**) HCT-8 cells after 24 hours. Results are the mean ± SD of six independent experiments performed in duplicate. * *p* < 0.05; ** *p* < 0.01; *** *p* < 0.001 *versus* CTRL.

Caco2 cells treated with GSP at the same concentration they have in 100 μg/mL of *Italia*, *Palieri* and *Red Globe* GSE, showed a significant growth inhibition with respect to untreated control (CTRL) at 24 hours (*p* < 0.001). EGC at the same concentration it has in 100 μg/mL of each GSE, had a minor inhibitory effect even though it is still significant (*p* < 0.01). The association EGC+GSP produced a statistically significant synergistic effect (Figure 2a). However, these inhibitory effects are significantly lower than those obtained with 100 μg/mL of the overall extract obtained from *Italia* and *Palieri* seeds (*p* < 0.001). In the contrast, GSP and the association EGC+GSP induced an inhibition rate on Caco2 cells which is higher than that has been observed with the extract from *Red Globe* seeds (Figure 2a).

HCT-8 cells treated with GSP at the same concentration they have in 100 μg/mL of *Italia* GSE showed a significant growth inhibition with respect to untreated control (CTRL) at 24 hours (*p* < 0.05). EGC at the same concentration it has in 100 μg/mL of *Italia* had no inhibitory effect, and the association EGC+GSP produced a statistically significant inhibitory effect, probably due to GSP contribution (Figure 2b). However, these inhibitory effects are significantly lower than those obtained with 100 μg/mL of the overall extract obtained from *Italia* seeds (*p* < 0.01).

HCT-8 cells treated separately with EGC and GSP at the same concentration they have in 100 μg/mL of *Palieri* and *Red Globe* GSE showed no growth inhibition with respect to untreated control (CTRL) at 24 hours, meanwhile the association EGC+GSP produced a statistically significant inhibitory effect with respect to CTRL (*p* < 0.05) (Figure 2b). It is noteworthy to point out that between *Palieri* and *Red Globe* GSE, only the treatment with the first GSE induced a statistically significant decrease in proliferation rate with respect to the EGC+GSP association.

**Figure 3:**
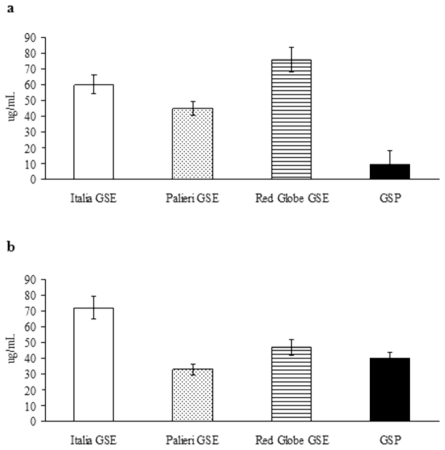
IC_50_ for GSE and GSP. (**a**) Growth inhibitory IC_50_ of *Italia*, *Palieri*, *Red Globe* GSE and GSP on Caco2 and (**b**) HCT-8 cells.

The growth inhibitory IC_50_ has been evaluated for the compounds under study, for weighing their relative anticancer efficacy (Figure 3). In Caco2 cells, growth inhibitory potency of GSP shows to be definitely greater than it has been recorded for individual GSE (Figure 3a); in contrast, in HCT-8 cancer cells, GSE obtained from *Palieri* cultivar exerts a higher growth inhibitory activity than GSP alone (Figure 3b). In both cell lines EGC showed only minor inhibitory activity (data not shown). These results confirm anticancer effects are tightly dependent on cancer cell differential sensitivity to tested compounds.

### 2.4. GSE-Induced Apoptosis

**Figure 4:**
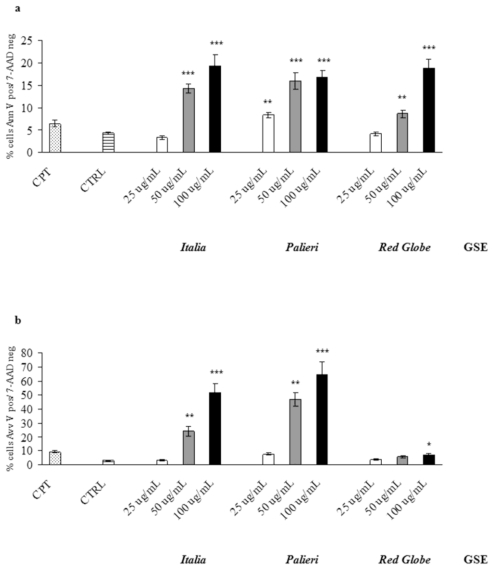
GSE-induced apoptosis. (**a**) Effects of *Italia*, *Palieri* and *Red Globe* GSE on apoptosis of Caco2 and (**b**) HCT-8 cells after 24 hours. Results are the mean ± SD of three independent experiments. * *p* < 0.05; ** *p* < 0.01; *** *p* < 0.001 *versus* CTRL.

GSE-induced apoptosis was evaluated by Annexin V and 7-AAD staining, where cells were treated with GSE (25, 50, 100 μg/mL) for 24 hours under similar conditions as in cell growth studies. Untreated (CTRL) and camptothecin-treated (CPT) cells were used as negative and positive controls respectively. In Caco2 cells *Italia*, *Palieri* and *Red Globe* GSE significantly induced apoptosis at 50 and 100 μg/mL. At 25 μg/mL, only *Palieri* GSE treated cells showed a statistically noteworthy increase in apoptotic rate. (Figure 4a).

In HCT-8 treated cells, the three cultivars significantly induced apoptotic cell death at the highest concentration (100 μg/mL), meanwhile only *Italia* and *Palieri* GSE induced a significant apoptosis at 50 μg/mL (Figure 4b).

### 2.5. Epigallocatechin and Procyanidin-Induced Apoptosis

**Figure 5:**
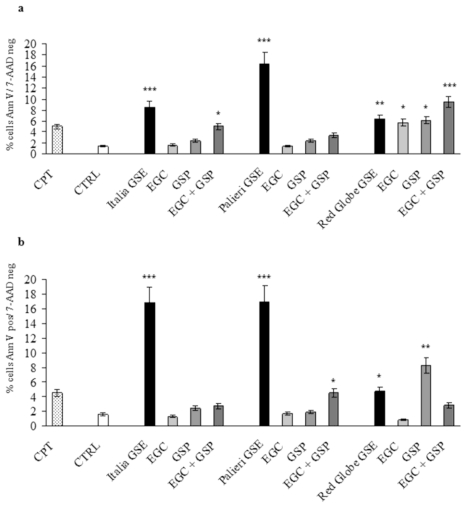
EGC and GSP induced apoptosis *versus* GSE. (**a**) Effects of commercially available EGC and GSP at the concentration they have in 100 μg/mL of GSE obtained from *Italia*, *Palieri* and *Red Globe* cultivars on apoptosis of Caco2 and (**b**) HCT-8 cells after 24 hours *versus* 100 μg/ml of GSE. Results are the mean ± SD of three independent experiments performed in duplicate. * *p* < 0.05; ** *p* < 0.01; *** *p* < 0.001 *versus* CTRL.

Apoptotic rate at 24 hours was evaluated in Caco2 and HCT-8 cells treated with EGC, GSP and EGC+GSP association at the same concentration they have in 100 μg/mL of each GSE. Untreated (CTRL) and camptothecin-treated (CPT) cells were used as negative and positive controls respectively. In both cell lines, EGC and GSP did not induce any relevant apoptotic effect. EGC+GSP association exerted a valuable apoptotic action on Caco2 cells only at the concentrations present in *Italia* GSE. Anyway, the apoptosis triggered by *Italia* GSE is significantly higher than that recorded with EGC+GSP association (*p* < 0.01) (Figure 5a).

In HCT-8 cells, EGC+GSP association exerted a valuable apoptotic action only at the concentrations present in *Palieri* and *Red Globe* GSE. Thus, the apoptosis triggered by *Palieri* and *Red Globe* GSE is significantly higher than has been recorded with EGC+GSP association (*p* < 0.001 and *p* < 0.01 respectively) (Figure 5b).

### 2.6. Immunoblot Analysis

Molecular parameters were evaluated only on samples treated with 50 μg/mL at 24 hours. This choice was suggested because cells treated with 100 μg/mL GSE underwent a massive apoptosis making it difficult to recover viable samples for immunoblot analysis.

#### 2.6.1. Caco2 Cells

**Figure 6:**
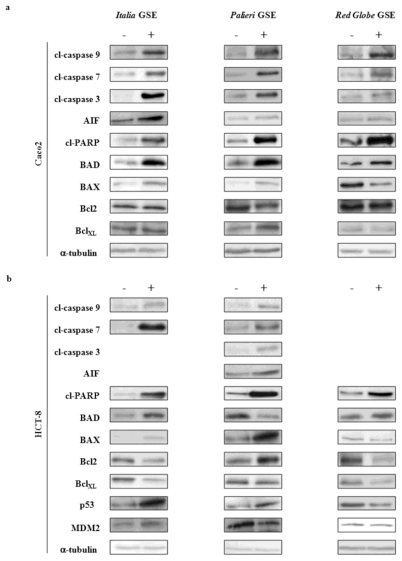
Immunoblot analysis. (**a**) Immunoblot showing the expression of cleaved caspases 9, 7 and 3, AIF, cleaved-PARP, BAD, BAX, Bcl2, BclXL, p53, MDM2 in Caco2 and (**b**) HCT-8 cells treated with 50 μg/mL of *Italia*, *Palieri* and *Red Globe* GSE for 24 hours. α-tubulin, served as a loading control.

At 24 hours, in *Italia*, *Palieri* and *Red Globe* treated samples, cleaved caspases 9, 7 and 3 were significantly increased (Figure 6a). A similar behavior was observed for AIF and cleaved-PARP. In addition, a significant increase in BAD and in both BAX/Bcl2 and BAX/BclXL ratios was observed in *Italia*, *Palieri* and *Red Globe* treated samples. p53 and p53/MDM2 ratio were not evaluated as Caco2 cells do not express p53 proteins.

#### 2.6.2. HCT-8 Cells

In *Italia* treated samples, caspases 9 and 7 and cleaved-PARP considerably increased at 24 hours (Figure 6b). A similar trend was observed for BAD, BAX/Bcl2, BAX/BclXL and p53/MDM2 ratios. Cleaved caspase 3 and AIF were not significantly modified with respect to control cells (data not shown).

In *Palieri* treated cells, activity of caspases 3, 7 and 9 increased significantly. Similarly, activity of both AIF and cleaved-PARP was increased by GSE treatment. The pro-apoptotic ratios of both BAX/BclXL and p53/MDM2 were augmented by GSE, meanwhile BAX/Bcl2 ratio was unmodified by treatment (data not shown). Paradoxically, BAD levels in *Palieri* treated cells appear to be significantly decreased.

In *Red Globe* treated samples, activity of caspases 9, 7 and 3 remained unchanged with respect to control cells, as well as AIF levels (data not shown); otherwise, cleaved-PARP, BAD, and both BAX/Bcl2 and BAX/BclXL ratios were significantly increased at 24 hours. p53/MDM2 ratio was unexpectedly, highly decreased.

GSE, which was obtained from the three cultivars, did not modify caspases-8 activity in both cell lines (data not shown). These data, assembled together, seem to suggest that GSE are likely to trigger apoptosis mainly through the intrinsic apoptotic pathways.

### 2.7. Discussion

In the last decade, several studies have documented the anticancer and cancer chemopreventive efficacy of GSE against various cancers, including human colon tumors [19,20]. These effects are usually attributed to the epigallocatechin and procyanidin content of the grape seed extracts. Indeed, catechins, epigallocatechin, epigallocatechin-3-gallate, as well as procyanidins share inhibitory and apoptotic properties. However, composition of grape seed extracts is highly complex, comprising several classes of active compounds. Therefore, it is likely that biological activity of GSE could be hardly be reduced only to its epigallocatechin and procyanidin content.

To address this question, we have firstly evaluated the anticancer effect exerted by GSE, which was obtained from three different cultivars, and subsequently we have compared this effect to that obtained by single commercially available compounds (EGC, GSP and the association of both), at the same concentration they have in 100 μg/mL of each seed extract. This choice was dictated by the fact that these compounds, largely known to be antiproliferative and pro-apoptotic, are routinely used in experimental work in the literature.

Our data provided evidence that the overall grape seed extract from *Italia* and *Palieri* cultivars induced growth inhibition and apoptosis in both Caco2 as well as in HCT-8 colon cancer cells; inhibition as well apoptotic rates induced by GSE were significantly greater than those recorded with EGC, GSP and the association of both. These data clearly support the hypothesis by which other flavan-3-ols are likely to participate to the overall GSE-mediated anticancer effect.

It is interesting to note, data which were obtained from treatment of both cell lines with *Red Globe* GSE were less effective. In fact, even though the overall GSE induced more apoptosis than single compounds, we did not find any decrease in growth rates in GSE-treated cells compared to cells treated with EGC and GSP alone and in association.

Hence, collectively, our results indicate that anticancer potency of GSE obtained from different cultivars exhibits some differences that cannot be explained only in terms of the relative epigallocatechin or procyanidin content, and other compounds seemingly can enhance (or even interfere with) the anticancer effects exerted by GSE. Indeed, it has been reported that GSE anticancer effects is linked to the pyrogallol-type structure [21], and to the presence of a gallate ester moiety at 3’ position of procyanidin B2 [22]. So far, differences in the relative concentration of compounds like these, could be related to their respective differences in biological activities.

Furthermore, GSE-related antineoplastic effects are highly specific for cancer type, as previously reported [23]. HCT-8 cells are differently sensitive to the anticancer effects triggered by GSE than Caco2 cells. This result is highlighted by comparing the IC_50_ obtained from the compounds under study. In Caco2 cells, GSP exert a relevant growth inhibitory activity, even at low concentration, significantly greater than that obtained with GSE. Otherwise, in HCT-8 cells, the highest growth inhibitory activity is recorded for *Palieri*-related extracts, whereas GSE obtained from *Red Globe* cultivar presented a comparable IC_50_ to that observed for GSP. EGC displayed only minor inhibitory activity.

GSE, extracted from different cultivars, seems to activate different apoptotic pathways, depending also on the specific cancer cell line under treatment. In Caco2 cells, both caspase-dependent as well as caspase-independent pathways are activated, through the involvement of AIF and caspase-3 as terminal effectors. Activation of AIF-dependent apoptosis during GSE treatment has been previously reported to occur in adrenal tumors and B-lymphoma [24,25]; however, AIF increase in GSE treated colon cancer cells is likely to be a specific feature of Caco2 [20], as no significant modification of AIF has been observed in HCT-8 cancer cells.

In *Palieri* and *Italia* HCT-8 treated cells, apoptosis is mainly triggered by caspases-3, meanwhile programmed cell death in *Red Globe* treated samples is likely to be dependent on the increase in both BAX/Bcl2 and BAX/BclXL apoptotic ratios.

## 3. Experimental Section

### 3.1. Cell Culture

The human colorectal cancer cell lines Caco2 and HCT-8 were obtained from European Collection of Cell Cultures (ECACC). Primary human fibroblasts were isolated from healthy dermis by a collagenase type II digestion.

Cells were seeded into 25-cm^2^ flasks (Falcon, Becton Dickinson Labware, Franklin Lakes, NJ, USA) in DMEM supplemented with 10% Fetal Calf Serum (FCS) and antibiotics (Penicillin 100 IU/mL, Streptomycin 100 μg/mL, Gentamycin 200 μg/mL). The cultures were kept at 37 °C in an atmosphere of 5% CO_2_ in air and the medium was changed every third day. At confluence, the cells were subcultured after removal with 0.05% trypsin-0.01% EDTA.

### 3.2. Sample Preparation and Analysis

*Italia* white grape, *Palieri* and *Red Globe* red grape cultivars from the experimental vineyard located in the Puglia region (Italy) were kindly provided by the Agricultural Research Council–Research Unit for grape and winegrowing in Mediterranean environment (CRA-UTV, Turi, BA, Italy). Fresh grape berry samples were skinned and seeds were separated from pulp and then the seeds were gently wiped with filter paper to eliminate pulp residues. Homogeneous and dry material was obtained from seeds, extracted with methanol, purified and analyzed by ESI-MS according to the previously published method [18]. GSE was resuspended in 70% ethanol at a concentration of 30 mg/mL and stored lightless at −20 °C until used. With the purpose of obtaining the concentration of 100 μg/mL (the highest concentration of GSE used in our experiments) GSE stock solutions were diluted 1:300.

EGC and GSP, provided by Sigma-Aldrich (St. Louis, MO, USA), were resuspended in 70% ethanol and used at the same concentration they have in the GSE of each cultivar to test growth inhibition and apoptosis. EGC and GSP were used at different concentrations with the aim to study their growth inhibitory potency (IC_50_) as isolated compounds.

### 3.3. Cell Proliferation Assay

Caco2, HCT-8 and human fibroblasts were seeded in 12-well culture plates (Falcon, Becton Dickinson Labware, Franklin Lakes, NJ, USA) at concentrations ranging between of 1 × 10^4^ cells/well and 3 × 10^4^ cells/well in a standard medium. After a zero time (T0) cell count, the cells were stimulated with 70% Ethanol (1:300, control), 25, 50, 100 μg/mL of *Italia*, *Palieri* and *Red Globe* GSE and incubated at 37 °C in an atmosphere of 5% CO_2_ in air. The cells were then detached from wells by trypsinization and cell count was performed by a cell counter (Beckman Coulter, Inc., Fullerton, CA, USA) and by a Thoma hemocytometer, after staining with the vital stain Trypan Blue (Sigma Chemical Co., St Louis, MO, USA) after 24, 48, 72 and 96 hours. Similar experiments were performed by stimulating cells with EGC and GSP at the same concentration they have in 100 μg/mL of each GSE for 24 hours. Two replicate wells were used for each data point, and every experiment was performed six times.

### 3.4. Apoptotic Cell Death Assay

Caco2 and HCT-8 cells were cultured at confluence into 25-cm^2^ flasks (Falcon, Becton Dickinson Labware, Franklin Lakes, NJ, USA) in a standard medium and stimulated with 70% Ethanol (1:300, control) and 25, 50, 100 μg/mL of *Italia*, *Palieri* and *Red Globe* GSE, EGC and GSP at the same concentration they have in 100 μg/mL of each GSE and incubated at 37 °C in an atmosphere of 5% CO_2_ in air. After 24 hours, the cells were trypsinized, washed twice with PBS and stained with FITC labelled Annexin V/7-AAD (7-aminoactinomycine-D) according to the manufacturer’s instructions (Instrumental Pro3 Laboratory, Cavenago, MI, Italy). The samples were then analyzed by flow cytometry (EPICS Coulter XL, Backman-Coulter Inc., Fullerton, CA, USA) for the quantification of apoptotic cells. The fluorescence of 20,000 events was measured and an excitation wavelength of 488 nm was used in combination with standard filters to discriminate between the FL1, FL3 channels, forward scatter and side scatter.

### 3.5. Immunoblot Analysis

Following 50 μg/mL GSE-treatment, Caco2 and HCT-8 cells were washed twice with ice-cold PBS and scraped in the following lysis buffer: 50 mM Tris-HCl, pH 7.4, 150 mM NaCl, 0.2% NP-40, 1% CHAPS, 2 mM EDTA dissolved in tetra-distilled water. A mix of protease inhibitors (Complete-Mini Protease Inhibitor Cocktail Tablets, Roche, Mannheim, Germany) was added prior to use. Cellular extracts were then sonicated and centrifuged at 14,000 rpm for 10 minutes. The protein content of supernatants was determined by using the Bradford assay. For immunoblot analyses, cellular extracts were separated on SDS-PAGE gels with a concentration of acrylamide, which is specific for the proteins studied. Proteins were blotted onto nitrocellulose membranes (BIO-RAD, Bio-Rad Laboratories, Hercules, CA, USA) and probed with the following antibodies: anti-AIF (sc-5586, Santa Cruz, CA, USA), anti-cleaved-PARP (Sigma, Saint Louis, MO, USA), anti-cleaved-caspase 9 (# 9501S, Cell Signaling Technology, Inc., Boston, MA, USA), anti-cleaved-caspase 7 (# 9491S, Cell Signaling Technology, Inc., Boston, MA, USA), anti-cleaved-caspase 3 (# 9661S, Cell Signaling Technology, Inc., Boston, MA, USA), anti-Bax (sc-493, Santa Cruz, CA, USA), anti-Bcl2 (sc-492, Santa Cruz, CA, USA), anti-BclXL (sc-8392, Santa Cruz, CA, USA), anti-p53 (sc-6243, Santa Cruz, CA, USA), anti-MDM2 (sc-5304, Santa Cruz, CA, USA), anti-BAD (sc-8044, Santa Cruz, CA, USA), anti-α-tubulin (Sigma, Saint Louis, MO, USA). Antigens were detected with an enhanced chemoluminescence (ECL) kit from Amersham (Amersham Biosciences, Little Chalfont, Backinghamshire, England) according to the manufacturer’s instructions.

### 3.6. Statistical Analysis

Data were expressed as mean ± SD and statistical analysis was performed through the analysis of variance (ANOVA), followed by the Bonferroni post-hoc tests. Differences were considered significant at the level of *p* < 0.05. Statistical analysis was performed by using GraphPad Instat software (GraphPad Software, Inc., San Diego, CA, USA).

## 4. Conclusions

Overall, these results highlight that GSE induces numerous and relevant anticancer effects, leading to growth arrest as well as to increased apoptotic rate in colon cancer cells. GSE exerts complex pleiotropic effects, depending on cancer cell types and on its molecular composition. Epigallocatechin and mainly procyanidins on their own display a relevant anticancer effect, but it is unlikely that the overall GSE dependent biological actions might be ascribed only to their content in grape seed extracts. Analytical studies reveal that several other polyphenolic compounds participate in the GSE composition. As a result, other factors probably synergize with epigallocatechin and procyanidins. Future studies are warranted in order to identify anticancer bioactives such as these described above.
